# Active management of third stage of labour, post–partum haemorrhage and maternal death rate in the Vanga Health Zone, Province of Bandundu, Democratic Republic of the Congo

**DOI:** 10.4102/phcfm.v2i1.76

**Published:** 2010-07-15

**Authors:** Jean-Pierre Fina Lubaki, Jean-Robert Musiti Ngolo, Lucie Zikudieka Maniati

**Affiliations:** 1 Vanga Hospital, Province of Bandundu, DR Congo; 2 Central Office of Vanga Health Zone, Province of Bandundu, DR Congo; 3 Maternal Child Health Integrated Program, Kinshasa, DR Congo

**Keywords:** active management of third stage of labour, Democratic Republic of the Congo, maternal death, post-partum haemorrhage, rural health

## Abstract

**Background:**

Post-partum haemorrhage (PPH) is the single largest cause of maternal death worldwide and a particular burden for developing countries. In Africa, about 33.9% of maternal deaths are due to PPH. In the Democratic Republic of the Congo (DRC), the prevalence of PPH is unknown. PPH can be prevented with active management of the third stage of labour (AMTSL).

**Objectives:**

To describe the practice of AMTSL in Vanga Health Zone and to calculate the incidence of PPH in Vanga Health Zone.

**Method:**

An intervention study with post-test-only design was conducted among health maternity wards using a data collection sheet to obtain information. All pregnant women attending Vanga Health maternity wards constituted the study population. Frequencies were determined for variables of interest.

**Results:**

From April 2007 to March 2008, 6339 deliveries took place at Vanga Health maternity wards, representing 71% of the institutional delivery rate. The number of deliveries realised with the practice of (AMTSL) were 5562; 366 cases of PPH were reported, making an incidence of 5.77%. Three cases of maternal deaths – two of which were related to PPH – were reported during the study period, which means there was a decline of 70% compared with the previous two years.

**Conclusion:**

The prevalence of PPH has been estimated to be 5.77%; PPH represents the cause of 67% of all maternal deaths. The extension of AMTSL practice, combined with the assurance of better supplies of oxytocin to enhance drug management, is strongly advised/suggested. As a number of births still take place outside the health maternity wards, the introduction of oral misoprostol could be considered a part of AMTSL for use by patients being treated by traditional midwives.

## INTRODUCTION

Post-partum haemorrhage (PPH) is the single largest cause of maternal death worldwide, being a particular burden for developing countries.^1, 2, 3^ In Africa, about 33.9% of maternal deaths are due to PPH.^3^ In the Democratic Republic of the Congo (DRC) the prevalence of PPH is unknown, but it is estimated that PPH accounts for about 16% of maternal mortalities.^3^ In the Vanga Health Zone, one of the 515 health zones in the DRC, the maternal mortality rate has been calculated to 152/100 000 live births in 2006 and 2007; the prevalence of PPH is unknown.^4, 5^

The most common definition of immediate PPH is blood loss of 500 mL or more in the first 24 hours following childbirth; severe PPH is defined as blood loss of 1000 mL or more.^6^ This definition is difficult to apply because some women, especially those who are anaemic, can develop hemodynamic disturbance with lower blood loss. In addition, an accurate estimation of the amount of blood loss during labour is difficult because in many rural settings only a visual assessment by the midwife is possible.

Every year, 14 million women experience PPH, and at least 128 000 of these women bleed to death.^7^ Uterine atony causes 60% of PPH.^8^ Two–thirds of PPH cases occur in women with no known risk factors.^9^

PPH can be prevented with AMTSL,^10^ which is the administration of a uterotonic within the first minute of birth, controlled cord traction and uterine massage.^11^ Oxytocin is the drug of choice considering its effectiveness, cost, side effects profile, interval for therapeutic effect and storage.^12^ A large WHO multicentre randomised controlled trial (RCT) found that oxytocin, in hospital settings, is the drug of choice.^13^

Four large-scale, randomised controlled trials that had compared active and expectant management of the third stage of labour found that AMTSL resulted in up to a 70% decrease in PPH and a decrease in the duration of the third stage.^14, 15, 16, 17^ The 2003 Cochrane Review found that AMTSL was associated with an approximate 60% reduction in the occurrence of PPH and severe PPH, a decreased need for blood transfusion, a decreased post–partum anaemia (Hgb < 9 g/dl), and an approximate 80% reduction in the use of therapeutic drugs.^18^

Based on the available evidence, the International Federation of Obstetricians and Gynecologists, and the International Confederation of Midwives, have agreed that the AMTSL should be offered to all women.^11^ Thadeus and Nangalia, in a study of the barriers to treatment of PPH, found that efforts to reduce the incidence of PPH as a major cause of maternal death must progress on two fronts: on the supply side, to ensure the provision of skilled care, and on the demand side, to ensure that women and their families accept the view that bleeding after birth is dangerous and that skilled care is preferable to traditional care.^19^

PPH is not listed as one of the indicators in the national system of health information in the DRC, which reflects that its importance is not recognised. After the conference on PPH in Entebbe, Uganda,^20^ the Vanga Health Zone, with the use of oxytocin as uterotonic, was the first in the DRC to implement AMTSL to prevent PPH in its health centres at the first trimester of 2007. The implementation of AMTSL leads to a system of monitoring PPH in Vanga Health Zone. A study was designed in order to describe the practice of AMTSL and to calculate the incidence of PPH in the setting.

## ETHICAL CONSIDERATIONS

The Health Ministry of the DRC has adopted the practice of AMTSL in 2008, and revised its directives for reproductive health. The study was conducted with the permission of the Vanga Health Zone authorities.

## METHOD

### Study design

The study was an intervention study with a post-test-only design among registered maternity centres of the Vanga Health Zone, in the Province of Bandundu, DRC. ^21^

### Study setting

The Vanga Health Zone is one of the 515 health zones in the Democratic Republic of the Congo. It is situated in the Province of Bandundu in the District of Kwilu. It covers an area of 2600 squared kilometres, with a population of about 222 612 habitants. Vanga is rural in its entire area with agriculture being the main source of revenue. The population is poor and many families are not able to pay hospital bills, even for a normal delivery. It is estimated that the average daily income is about $0.50.

The Vanga Health Zone has a general hospital and 48 peripheral health maternity wards. The furthermost health maternity ward of the Vanga Health Zone is about 90 kilometres from the hospital. Seven maternity wards have installed systems that provide electricity but only one of them (in the general hospital) seems to be reliable. The other systems were not functioning at the time of study. Routes of communication are in a disastrous state. The health centres are managed by nurses and deliveries are supervised by trained midwives.

The Vanga Health Zone has an average institutional delivery rate of about 77% and the maternal mortality has been calculated to 152/100 000 births in 2006 and 2007.^4, 5^

### Study population

All pregnant women who attended the Vanga Health Zone maternity wards represented the study population. Approximately 6339 pregnant women took part.

### Data collection

A one-week training session on PPH was organised for all midwives working in the 48 health maternity wards.

A data collection sheet, with all the information necessary to assess the variables of the study, was designed and copies were sent to all maternity wards to be filled out monthly by the midwives. Once the sheets were completed they were sent to the Central Office of the Vanga Health Zone so that the data could be entered into a computer. To enhance the validity of the information, a supervised data–quality audit was performed during the study period. The duration of the study was one year, from 01 April 2007 to 30 March 2008.

Each month, 40 sheets were received from the health centres and hospital maternities.^5^

### Variables

- Number of institutional deliveries.
- Number of institutional deliveries with AMTSL.
- Number of cases PPH.
- Number of cases of maternal death.

### Analysis

Data gathered from the health maternity wards were entered in the computer. Frequencies have been calculated for key variables of interest.

### Bias

Biases present in this study can be identified as: ^22^

- Observer bias, due to the fact that the definition of PPH is largely subjective. The training of midwives could be a way of minimising this bias.
- Selection bias, due to the fact that not all women were delivering in health maternity wards. The researchers supposed that the Vanga Health Zone had a high institutional delivery rate and that the segment of the pregnant women attending the health maternity wards could therefore be regarded as being a representative example of the characteristics of the wider population of interest.

## RESULTS

The study took place from 01 April 2007 to 30 March 2008. During this period, 576 data collection sheets were received during the study period from the 48 health maternities. Of these, 531 data collection sheets came from health centres, representing a proportion of 92%. Table 1 summarises the information gathered from the data collection sheets.

**TABLE 1:** Distribution of deliveries in Vanga Health zone

Setting	Quantity			Outcome	
	
	Institutional deliveries	Institutional deliveries with AMSTL	Cases of PPH	Cases of PPH	

				Stop	Death
Vanga health Zone	6339	5562	366	364	2

### Institutional deliveries

With a total population of 222 612 inhabitants, it was estimated that 8 904 deliveries were expected during the study period in the Vanga Health Zone. There were 6339 deliveries registered in the 48 maternities, representing an institutional delivery rate of 71%. Of these deliveries, 293 were caesarean sections, constituting 4.66% of institutional deliveries.

### Active management of third stage of labour

AMTSL was practiced in 5562 of the deliveries, representing a proportion of 88% of the total 6339 deliveries that took place in the maternities.

### Post-partum haemorrhage

Three hundred and sixty-six cases of PPH were reported from the maternities, constituting a proportion of 5.77% of all the deliveries. Three hundred and sixty-four cases of PPH were resolved after treatment; two cases resulted in death. Six cases of PPH were referred by the health centres to the general hospital, representing a proportion of 1.6%.

### Maternal death

Three maternal deaths were reported during the study period in the Vanga Health Zone, two of which were due to PPH. One of the six cases of PPH that was referred by health centres to the general hospital subsequently died of severe anaemia. Compared with the two previous years, in which 10 cases of maternal deaths were reported annually, the results noted a decrease of 70% in maternal deaths.

## DISCUSSION

The Vanga Health Zone is one of the older pilot health zones of the DRC, where the participation of the population in sustaining health structures is one of the best in the country. It has a high institutional delivery rate of 71% compared with national average of 42%. This institutional delivery rate is similar to what was found in a study in a rural district of Zimbabwe (75%) but higher compared to that found in rural Nigeria (51%).^23, 24^ In Vanga, the caesarean section rate was 4.66%, while it was estimated to 6.3% in the previous cited study in Zimbabwe and 9.1% in a study in Ghana, Nigeria.^23, 25^ Indications for the caesarean sections have not been assessed in our study. Misoprostol has been recommended for use in settings where the necessary supplies are not available to give an injection of oxytocin.^26^ In our context, even if the majority of health centres have no electricity, it is assumed that oxytocin can be administered without major problems.

Many health centres, ranging from 5.5% – 8.52% quarterly, ran out of oxytocin during the survey,^27^ so that the drug coverage of AMTSL was 88% of institutional deliveries. Extending the coverage of AMTSL in Vanga health zone could be achieved through better supply of oxytocin to health centres.

The identification of PPH by means of visual recognition of abnormally-excessive bleeding after birth is largely dependent upon the experience of practitioners. Therefore, the results of the study could have been influenced, and even compromised, by the under- or over-reporting by practitioners. Prendiville, Elbourne and McDonald found that clinical estimates of blood loss are generally thought to be underestimated by 35% – 50%.^18^ In clinical trials of oxytocin in AMTSL, the occurrence of PPH has been fewer than in this study,^13, 28^ highlighting the gap that exists between research and reality.

Three maternal deaths were reported during the study. One may note that the pregnant women were surveyed only when attending the maternity for delivery and not during the pregnancy, which represented a limitation in assessing the maternal mortality rate. Nevertheless, a dramatic decrease of maternal deaths was noted.

## References

[1] AbouZahr C. Global burden of maternal death and disability. British Medical Bulletin. 2003;67:1–11. 14711750 10.1093/bmb/ldg015

[2] World Health Organization Maternal Mortality: A Global Factbook. Geneva: WHO; 1991.

[3] Khan KS, Wojdyla D, Say L, Gülmezoglu AM, Van Look PFA. WHO analysis of causes of maternal death: A systematic review. Lancet. 2006;367:1066–1074. 16581405 10.1016/S0140-6736(06)68397-9

[4] Zone de santé rurale de Vanga Rapport annuel 2006. Vanga: ZSV; 2007.

[5] Zone de santé rurale de Vanga Rapport annuel 2007. Vanga: ZSV; 2008.

[6] Prendiville WJ, Elbourne D. Care during the third stage of labor In: Chalmers I, Enkin M, Keirse MJNC, editors. Effective Care in Pregnancy and Childbirth. Oxford: Oxford University Press, 1998; p. 1145–1169.

[7] World Health Organization (WHO) Mother-Baby Package: Implementing Safe Motherhood in Countries. Geneva: WHO; 1998.

[8] AbouZahr Antepartum and post–partum haemorrhage In: Murray CJL, Lopez AD, editors. Health dimensions of sex and reproduction: The global burden of sexually transmitted diseases, maternal conditions, perinatal disorders, and congenital anomalies. Geneva: WHO, 1998; p. 165–190.

[9] Akins S. Postpartum haemorrhage. A 90s approach to an age-old problem. J Nurse Midwifery. 1994;39:123S–134S. 8035242 10.1016/0091-2182(94)90070-1

[10] McCormick M, Sanghvi H, Kinzie B, McIntosh N. Preventing postpartum hemorrhage in low–resource settings. Int J Gynaecol Obstet. 2002;77:267–275. 12065142 10.1016/s0020-7292(02)00020-6

[11] FIGO ICM Joint Statement Management of third stage of labour to prevent post-partum Haemorrhage. 2003. 10.1016/j.jmwh.2003.11.005 14710151

[12] WHO recommendations for the prevention of postpartum haemorrhage. Geneva: WHO; 2007.

[13] Gülmezoglu AM et al. WHO multicentre randomised trial of misoprostol in the management of the third stage of labour. Lancet. 2001;358:689–695. 11551574 10.1016/s0140-6736(01)05835-4

[14] Bagley C. A comparison of active and expectant management of the third stage of labour. Midwifery. 1990;6:3–27. 2182978 10.1016/s0266-6138(05)80091-9

[15] Khan GQ. Controlled cord traction versus minimal interventions techniques in delivery of the placenta: A randomized controlled trial. Am J Obstet Gynecol. 1997;177:770–774. 9369817 10.1016/s0002-9378(97)70266-7

[16] Prendiville WJ, Elbourne D, Chalmers I. The effects of routine oxytocic administration in the management of third stage of labour: An overview of the evidence from controlled trials. Br J Obstet Gynaec. 1998;95:3–16. 10.1111/j.1471-0528.1988.tb06475.x 3277663

[17] Rogers J, Wood J, McCandlish et al. Active versus expectant management of third stage of labour: The Hinchingbrooke randomised controlled trial. Lancet. 1998;351:693–699. 9504513 10.1016/S0140-6736(97)09409-9

[18] Prendiville W, Elbourne D, McDonald S. 2003 Active versus expectant management of the third stage of labour. [Cochrane Review] In: The Cochrane Library, Issue 3, 2003. Chichester, Update Software.

[19] Thaddeus S, Nangalia R. Perceptions Matter: Barriers to treatment of post-partum haemorrhage. J Midwifery Womens Health. 2004;49(4):293–297. 15236708 10.1016/j.jmwh.2004.04.010

[20] Sanghvi H, Lewison D, editors. Report of a conference in Entebbe. Preventing Mortality from post partum hemorrhage in Africa: Moving from Research to Practice; 2006 April 4-7; Entebbe, Uganda JHPIEGO, Baltimore; 2006.

[21] Leedy P. Practical research: planning and design. Columbus: Merrill; 1997.

[22] Bowling A. Research methods in health: Investigating health and health services Oxford: Oxford University Press; 2002.

[23] Nilses C, Nyström L, Munjanja S, Lindmark G. Self-reported reproductive outcome and implications in relation to use of care in women in rural Zimbabwe. Acta Obstet Gynecol Scand. 2002;81(6):508–515. 12047303 10.1034/j.1600-0412.2002.810606.x

[24] Nwakoby BN. Use of obstetric services in rural Nigeria. J R Soc Health. 1994;114(3):132–136. 7932482 10.1177/146642409411400304

[25] Martey JO, Djan JO, Twum S, Browne EN, Opoku SA. Maternal mortality due to post-partum haemorrhage. Int J Gynaecol Obstet. 1993;42(3):237–241. 7901078 10.1016/0020-7292(93)90217-k

[26] Goldberg AB, Greenberg MA, Darney PD. Misoprostol and pregnancy. N Engl J Med. 2001;344:38–47. 11136959 10.1056/NEJM200101043440107

[27] Vanga Post-Partum haemorrhage Project Monitoring and evaluation report. Vanga: VPPHP; 2008.

[28] Oboro V, Tabowei TO. A randomised controlled trial of misoprostol versus oxytocin in the active management of the third stage of labour. J Obstet Gynaecol. 2003 1;23(1):13–16. 12623474 10.1080/0144361021000043146

